# Dr. Frank Guy Sherwood

**Published:** 1911-03

**Authors:** 


Dr. Frank Guy Sherwood, of Albion, N. Y., died January 25, 1911, from pleuro-pneumonia, aged 60 years. He received his degree in medicine from the University of Buffalo, in 1877.

